# Equip your vehicle for the right terrain: Using features of local pathology to guide engineering of targeted drug delivery systems

**DOI:** 10.1016/j.jconrel.2025.114412

**Published:** 2025-11-15

**Authors:** Taylor V. Brysgel, Jing Liu, Elizabeth D. Hood, Jacob S. Brenner, Vladimir R. Muzykantov

**Affiliations:** a Department of Systems Pharmacology and Translational Therapeutics, Perelman School of Medicine, University of Pennsylvania, Philadelphia, PA 19104, USA; b Division of Pulmonary, Allergy, and Critical Care, Department of Medicine, Perelman School of Medicine, University of Pennsylvania, Philadelphia, PA, USA

**Keywords:** Drug delivery systems, Nanoparticles, Endothelium, Drug targeting, Vascular inflammation

## Abstract

Drug delivery systems (DDS) have been engineered to target therapeutics to the primary site of disease for many varied pathologies. Lung disorders are especially advantaged, as the pulmonary endothelium’s direct interface with the bloodstream makes it accessible and highly susceptible to injury. As such, the endothelium is damaged in many pathologies, characterized by dysregulation and structural and functional changes. Over the years, scientists have exploited these key features of endothelial dysfunction for targeted drug delivery. This review combines past and present research on DDS design directed toward the pathological pulmonary endothelium. Scientists have engineered DDS for endothelial delivery through the modification of nanocarrier size, shape, rigidity, and surface features, and the use of ligand-directed, biomimetic, stimuli-responsive, and physicochemical targeting strategies. While recent advances have brought these approaches closer to clinical relevance, challenges remain in their translation to therapeutic applications.

## 1. Introduction: vessel structure in the macro- *vs* micro-vasculature

The vascular endothelium is a thin, continuous monolayer of endothelial cells (EC) that line the inner surface of the circulatory system, including arteries, arterioles, capillaries, venules, and veins [1]. This highly specialized cell layer forms a regulatory interface between the bloodstream and surrounding tissues. Remarkably, the total surface area of the endothelium in the human body is estimated to be between 3000 and 6000 square meters, roughly the size of an American football field, and consists of 1 to 6 × 10^13^ endothelial cells [2]. This expansive cellular network provides a vast surface area for molecular and cellular interactions within the vascular system [3].

**Fig. 1.**
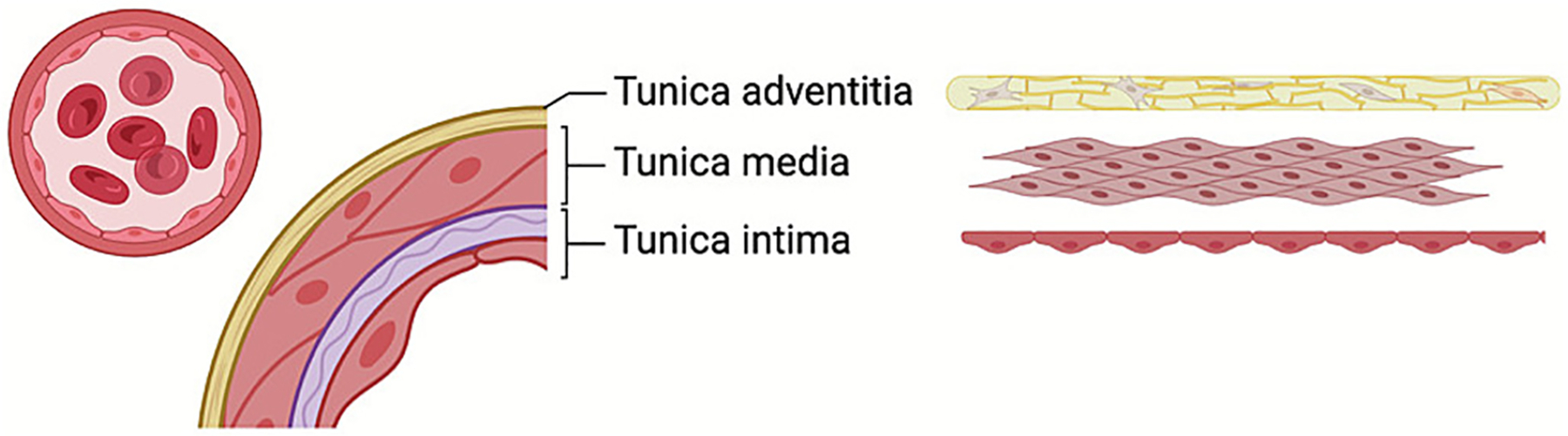
Generic structure of the macro-vasculature. This magnified image shows three layers in an arterial vessel: tunica intima (consisting of a tight endothelial monolayer lining the basal membrane), tunica media (predominantly composed of smooth muscle cells) and tunica adventitia (consisting mostly of connective tissue and the vasa vasorum). Created in BioRender. Brenner, J. (2025) https://BioRender.com/u6cl422

Based on vessel lumen diameter and function, the vascular system is broadly divided into the macro-vasculature (approx. >0.1 mm) and micro-vasculature (~8 μm to 0.1 mm) [4]. Macro-vascular vessels, such as the aorta, carotid arteries, and large veins, are composed of three concentric layers: the tunica intima, tunica media, and tunica adventitia (Fig. 1) [5]. The innermost layer directly in contact with blood flow is the tunica intima, which consists of a monolayer of ECs supported by a thin layer of the basal membrane containing collagen type IV, fibronectin, laminin, and other components of elastic tissue [6]. The second vascular layer is the tunica media, which is found primarily in arteries and veins and is composed of transversely arranged smooth muscle cells enveloped in elastic fibers [5]. The outermost layer, tunica adventitia, contains relatively loose large collagen fibrils, which connect the vessel with surrounding tissues [7].

At the highest level, the macro-vasculature serves as a conduit that distributes blood efficiently throughout the body. While it is built to withstand high blood pressure, this environment creates a major barrier to molecular transport [8]. The macro-vasculature experiences high shear stress (>10–70 dynes/cm^2^) and violent, pulsatile blood flow [9]. As a result, the primary functional goal of endothelial cells in these vessels is to survive and maintain the structure of the vessel, rather than to facilitate trans-vascular transport. Furthermore, the thick tunica media and large number of smooth muscle cells make transport deep into the tissue of the macro-vasculature difficult [10]. These factors severely limit the ability of macromolecules, proteins, or drug delivery systems (DDS) to bind with ECs in the macro-vasculature, let alone get internalized. Consequently, targeting large vessels from the luminal side is inefficient and generally ineffective for most therapeutic applications.

In contrast, the micro-vasculature, which includes arterioles, capillaries, and venules, are specialized for fine-tuned regulation at the tissue level. Capillaries are composed of just one cellular layer, ECs forming the tunica intima [5]. These ECs are continuous and lack any openings (fenestrae) into their cytoplasm [11]. They are held together by a series of tight junctions composed of occludins, claudins, and junctional adhesion molecules that bring the plasma membrane of ECs together, forming a nearly impermeable wall [12]. Vascular endothelial cadherin (VE-cadherin), platelet endothelial cell adhesion molecule 1 (PECAM1), intercellular adhesion molecule 1 (ICAM1), and claudin-5 are a few prominent pan-endothelial adhesion proteins that maintain the integrity of the endothelium across the vasculature [13]. Due to its direct exposure to circulating molecules, solutes, immune and other cells, and pathogens, ECs serve as first-line sensors and regulators of vascular functions. These include monitoring vascular tone and blood pressure and regulating permeability, the immune system, coagulation, and hemostasis [14,15].

In this review, we will explore the features of the micro-vasculature, specifically the pulmonary micro-vasculature, that make it a viable target for nanocarrier (NC) based DDS. Notably, we will look at features of the dysregulated endothelium, which is present in several pathologies, that can be exploited by researchers designing drug delivery systems to enhance therapeutic delivery. Effective drug delivery to the dysregulated endothelium is essential for restoring vascular homeostasis and improving outcomes across a range of inflammatory and ischemic diseases, and we hope to highlight the many years of research that have gone into advancing this field.

### 1.1. Functions of the pulmonary endothelium

**Fig. 2.**
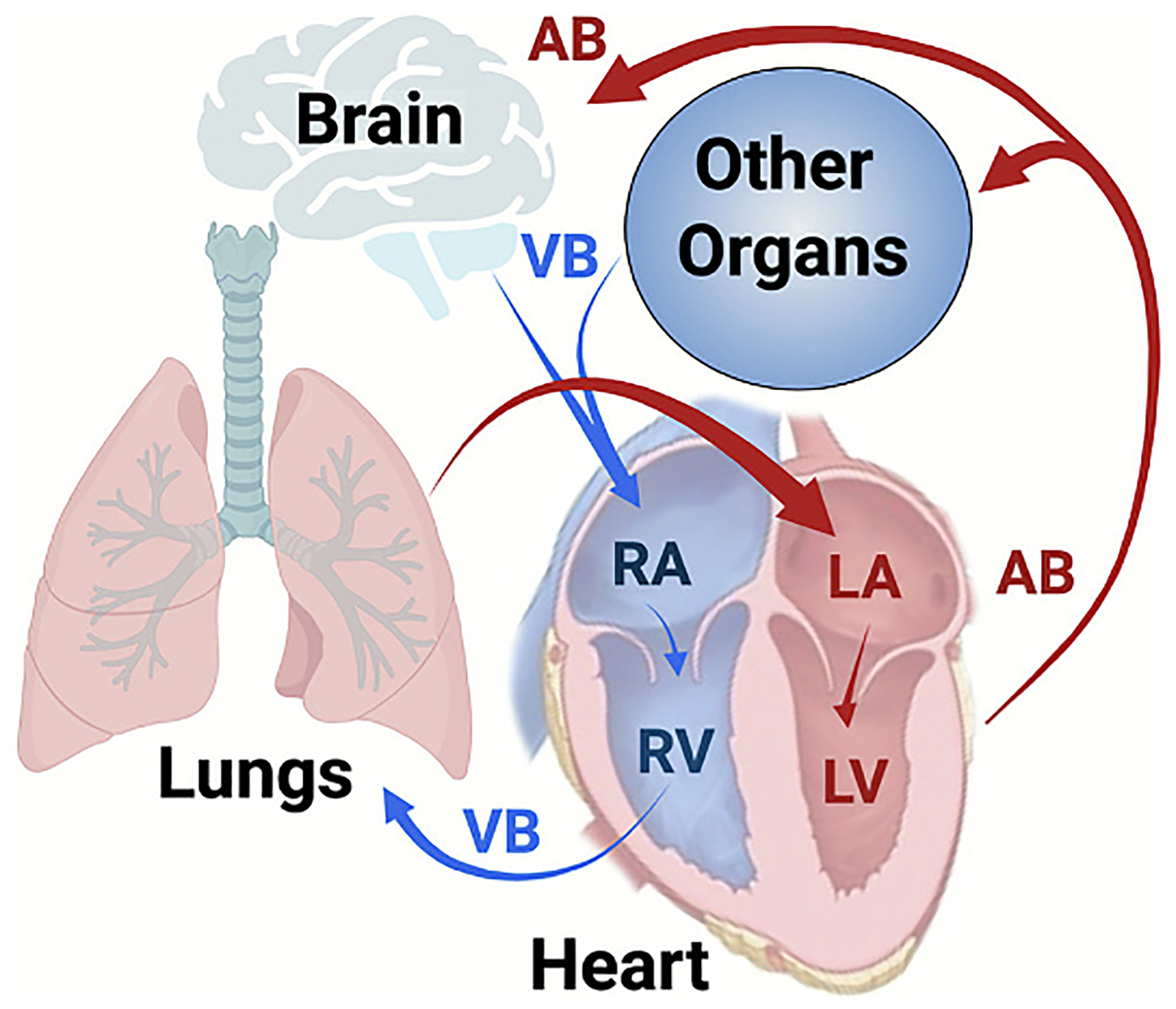
Deoxygenated blood enters the right atrium of the heart, then moves to the right ventricle, and is pumped to the lungs via the pulmonary artery. In the lungs, the blood is oxygenated, filtered, and several enzymatic processes take place. Blue arrows designate venous blood flow (VB), and red arrows arterial blood (AB). Image created in BioRender. Brenner, J. (2025) https://BioRender.com/u6cl422

Lungs perform several vitally important functions. First, they oxygenate blood and remove carbon dioxide *via* gas exchange between alveolae and the capillary meshwork surrounding these terminal airway structures [16] (Fig. 2). In addition to this negotiation enabled by reversible interactions of gases with hemoglobin packed in red blood cells (RBCs), the lungs exert several less appreciated yet important roles. These include the catalytic revitalization of venous blood mediated by numerous peptidases and other hydrolytic enzymes populating the luminal surface. For example, angiotensin converting enzyme (ACE) generates angiotensin II, regulating both vasodilatory and vasocon-strictive processes, and CD39 ATPase removes platelet activator ADP, thereby modifying clot formation [17,18]. Suppression of this blood-cleaning activity leads to the surge of vasoactive peptides in the blood which promotes hypertension, vascular edema, and inflammation, among other abnormalities [19].

Additionally, pulmonary microcirculation serves as a filter that mechanically retains small thrombi, rigidified senescent RBCs, and aggregates composed of abnormal cells or subcellular components (exosomes). Failure of this filtration can lead to contaminated blood entering circulation, leading to microembolisms in the brain and other organs with sensitive and fragile vasculature [20]. Surprisingly, the pulmonary micro-vasculature also plays a critically important role in the genesis of platelets [21]. Moreover, the pulmonary micro-vasculature hosts marginated leukocytes (immune cells that reside in the lungs), which are activated and vacate the lungs in response to pro-inflammatory mediators in blood [21].

### 1.2. Targeting DDS to the pulmonary endothelium

The pulmonary endothelium is an attractive and strategic target for drug delivery due to its vast surface area and unique physiological characteristics. The pulmonary endothelium represents the body’s largest vascular interface encompassing about one-third of the entire vascular surface, ~130 sq. meters in adults [22]. This vast expanse ensures widespread exposure of circulating DDS to endothelial cells, enhancing the probability of interaction and uptake. In addition to its size, the pulmonary endothelium expresses a rich array of surface proteins suitable for targeted delivery. These include the various cell adhesion molecules (CAMs) and cadherins, as well as numerous pattern recognition receptors (PRRs), particularly toll-like receptors (TLRs), which are of high interest for immune-targeted NCs [23]. This molecular diversity offers numerous options for ligand-mediated targeting.

Another key advantage is the lung’s central role in systemic blood flow. The lungs receive the entire cardiac output from the right ventricle, equivalent to the systemic output of the left ventricle, making the pulmonary endothelium the first vascular bed encountered by intravenously injected DDS [24] (Fig. 2). This “first-pass” exposure ensures that pulmonary ECs are the initial and most frequent points of contact for therapeutic carriers. The high perfusion, low-resistance flow in pulmonary capillaries further favors intimate interactions between DDS and endothelial cells, optimizing the potential for both passive and active targeting [25].

The pulmonary endothelium possesses specialized cellular machinery that facilitates the uptake of NCs. ECs are equipped with robust endocytic systems, including caveolae and clathrin-coated pits, which can internalize even large immunoconjugates circulating in the bloodstream [26]. Receptor-mediated endocytosis, especially clathrin-mediated endocytosis (CME), plays a pivotal role in the uptake of large molecules, with clathrin-adaptor proteins and actin filaments driving the formation and invagination of vesicles [27]. Caveolin-coated vesicles, in particular, enable transcytosis of large therapeutic agents from the vascular lumen to interstitial compartments *via* intracellular trafficking [28]. While paracellular transport is also possible, it is significantly restricted by tight junctions between healthy ECs [29].

## 2. Design considerations for DDS targeting the pulmonary endothelium

When designing NCs targeting the pulmonary endothelium, there are several features that need to be carefully considered. Notably, the size, shape, and flexibility of the DDS, together with the characteristics of surface targeting ligands (*e.g*., affinity, avidity, and ligand density) can collectively influence *in vivo* behavior [30]. These physicochemical parameters can alter the targeting, cellular uptake, and clearance profiles of a DDS. Briefly, we will go into more details on each of these factors:

### 2.1. Passive (non-affinity) NC-based delivery

#### Size and shape:

NC size is a critical determinant of its circulation time, pharmacokinetics, biodistribution, clearance, and organ-specific targeting [31,32]. For example, NCs smaller than 10 nm are typically cleared rapidly *via* renal filtration or through non-specific trans-endothelial transport [33]. In contrast, particles larger than 200 nm are often sequestered by the mononuclear phagocyte system (MPS), particularly in the liver, spleen, and bone marrow, leading to reduced circulation time [31,32]. Micron-sized particles (20–50 μm) may become mechanically entrapped in the micro-vasculature, especially in the pulmonary capillaries and precapillary arterioles, resulting in passive localization [34].

NC shape is another fundamental property which influences NC behavior in circulation and interactions with other biological systems [35,36]. For example, elongated carriers, such as nanorods, elliptical disks, and filomicelles, exhibit prolonged circulation times, reduced uptake by phagocytic immune cells, and enhanced lateral migration compared with spheres [37–40]. Under shear stress, nanorods display a higher binding probability due to their lateral margination and extended contact with the endothelium [41]. This is because an elongated geometry facilitates rolling and tumbling motions under flow, enhancing interactions with the vessel wall and promoting passive localization. Computational and microfluidic studies further indicate that non-spherical microparticles (ie, oblate or discoid NCs >500 nm) adhere more effectively to the vascular endothelium [42]. Simulations comparing cones, cubes, rods, rice-shaped, and sphere NCs show that geometry also modulates intracellular trafficking and translocation [43–45].

#### Flexibility.

*In vitro* studies have demonstrated that rigid particles are five times more likely to be internalized by immune cells compared to their flexible counterparts, which extends circulation and increases their chances of interacting with endothelial targets [35]. In addition, flexibility allows NCs to deform and traverse narrow vessels, thereby avoiding mechanical entrapment within the micro-vasculature [46]. This advantage is especially evident when compared to rigid, elongated particles, such as rods, disks, and tubes, with dimensions exceeding several hundred nanometers, as these particles are more likely to get lodged in small capillaries [35]. In this context, RBCs represent a natural model for an optimal DDS due to their remarkable elasticity. RBCs have a diameter of ~7 μm, yet repeatedly deform to pass through capillaries of ~2 μm while evading immune clearance [47]. Thus, some synthetic DDS (*e.g*., lipobeads and hydrogels) are engineered to mimic the key features of RBCs, notably their mechanical flexibility, for enhanced drug delivery [48,49].

#### Hemodynamics.

Hemodynamic factors such as blood flow, shear stress, and fluid mechanical forces can dramatically influence how NCs interact with and bind to the vascular endothelium [50]. Simulation and microfluidic studies have shown that smaller NCs migrate to vessel walls more rapidly than larger ones, likely due to higher diffusion coefficients, which are inversely proportional to particle size [51–53]. However, some flow chamber studies have shown that polystyrene nanoparticles larger than 200 nm can also effectively migrate toward vessel walls, driven by sedimentation in horizontal capillaries and pronounced lateral drift in vertically oriented capillaries with downward flow [54].

#### 2.1.1. Passive lung accumulation and intracellular delivery—advantages and limitations

**Fig. 3.**
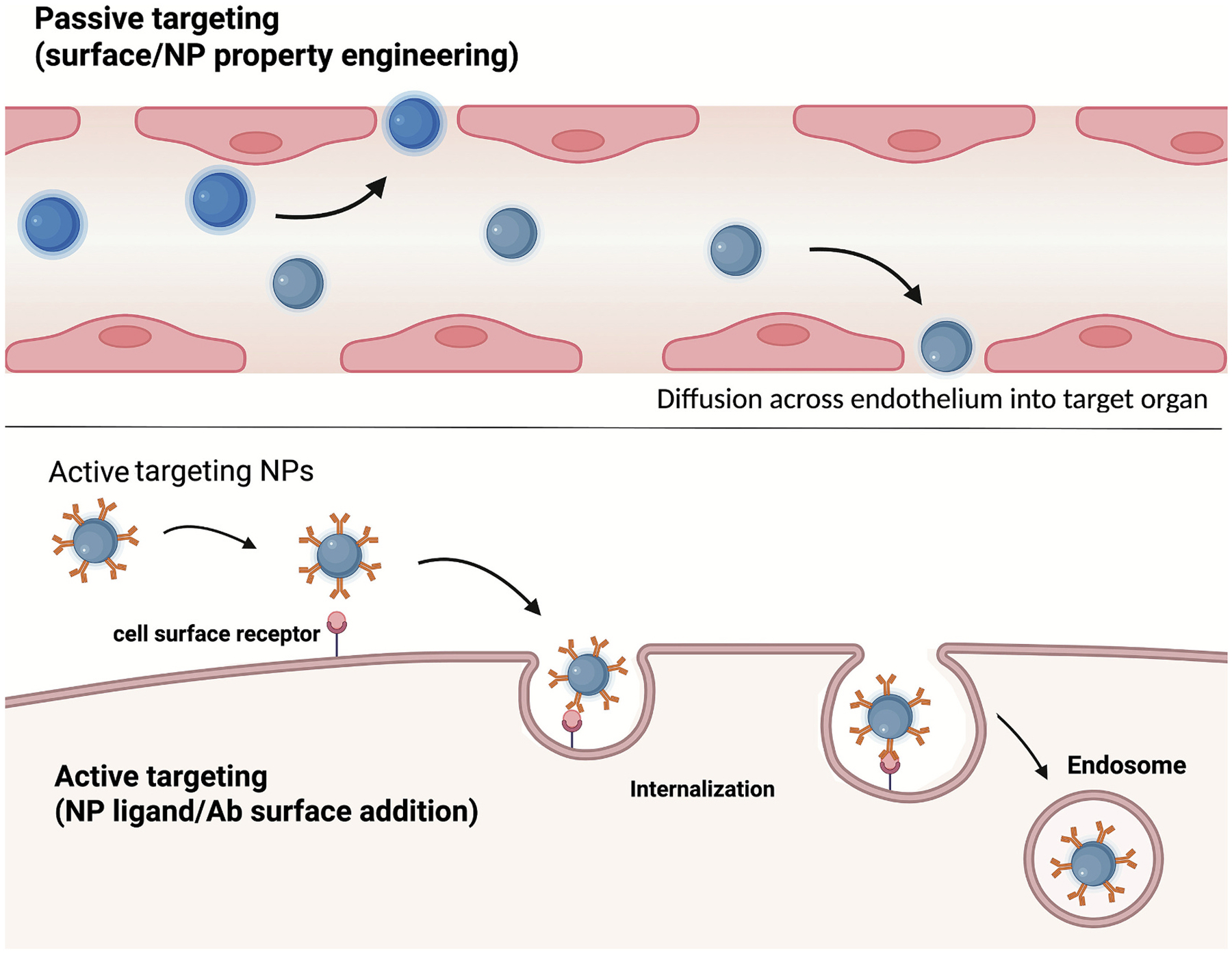
Active vs Passive Targeting of Nanocarriers: For passive targeting, the surface and engineered properties of the NC drive diffusion across the endothelium into the target organ. In active targeting, NCs are modified with targeting ligands (antibodies shown here) that bind to cell surface receptors, leading to cellular internalization via a variety of mechanisms. Created in BioRender. Brenner, J. (2025) https://BioRender.com/u6cl422

Passive targeting of DDS relies on the physical features of the tissue microvasculature, the nanocarrier, and the administration route, directing DDS delivery to a diverse array of organs and tissues [46,55,56]. For example, particles ~10–200 nm tend to accumulate in inflamed tissue due to the enhanced permeability of the pathological vasculature (Fig. 3). In the lungs, passive localization of non-affinity-based DDS is mainly driven by mechanical entrapment in the pulmonary microvascular bed following intravenous injection. NCs with micron-scale size and low deformability are especially prone to this form of physical sequestration [57]. For example, albumin particles (~10 μm) show significant lung entrapment due to mechanical filtration in narrow pulmonary capillaries [46,57]. Similarly, non-deformable polystyrene NCs (~500 nm) demonstrate lung retention in rodent models independent of any active targeting mechanism [58,59]. By exploiting these passive processes, drug-loaded NCs can enhance lung-targeted delivery after a single dose, thereby improving site-specific efficacy [55].

However, passive targeting has its limitations. Since passive accumulation depends on vascular physiology, tissue heterogeneity can produce uneven drug distribution across regions [46,55]. Passive retention likewise offers limited cell-type specific targeting [46]. In the lungs, this size-dependent entrapment poses significant risks, including microvascular occlusion, embolization, impaired tissue perfusion, and rapid clearance by macrophages [46,59]. These effects collectively reduce both circulation time and targeting efficiency. More broadly, passive sequestration lacks cell specificity, which compromises the efficacy of the therapeutic [34,46,60].

It is important to remember that the intracellular delivery of non-affinity DDS is mainly through non-specific endocytosis, phagocytosis, or micropinocytosis [60]. Therefore, engineering controlled, site-specific cargo release to specific cellular compartments, such as cytosol, nucleus, or mitochondria, while avoiding lysosomal degradation is crucial to maximize efficacy and reduce off-target effects [61]. Numerous bio-inspired NCs achieve this using stimuli-responsive chemistries and materials that couple targeting with on-demand payload release. For example, pH-sensitive materials trigger cargo release in an acidic environment (pH ~5–6), promoting endosomal escape and cytosolic delivery before lysosomal degradation [62]. In reactive oxidative species (ROS) rich microenvironments, such as inflamed lung tissue, redox-responsive NCs can be employed to trigger on-site payload release in response to redox changes, concentrating drug action at disease sites [62]. Other platforms use exogenous triggers, such as light, for precise spatiotemporal control [63]. Collectively, optimizing particle design for controlled release, cell-type specificity, and intracellular trafficking is essential for the therapeutic translation of lung-targeted DDS.

### 2.2. Active (affinity-based) targeted delivery systems

#### Size and shape.

Size also plays a pivotal role in modulating the efficacy and specificity of ligand or antibody-targeted NCs, as it can influence endothelial cell internalization, the route of endocytosis, and cell-type specificity [64]. In general, anti-CAM coated NCs (~100–300 nm) can be effectively internalized *via* CAM-mediated endocytosis (Fig. 3); however small changes in NC size can have a significant impact on their internalization. Larger (~300 nm) nanocarriers have enhanced binding to endothelial cells due to their greater surface area, but smaller (~100 nm) nanocarriers are more readily endocytosed [64]. For example, anti-ICAM1 conjugated DDS are efficiently internalized at ~100–200 nm but not when they are ≥1 μm [65]. Consequently, anti-ICAM1/tissue plasminogen activator (tPa) conjugates of ~1 μm, are primarily retained on the pulmonary endothelial surface, thereby augmenting intravascular fibrinolysis [65].

Similarly, plasmalemmal vesicle associated protein 1 (PV1) targeted NCs smaller than 50 nm are efficiently internalized by endothelial cells through caveolae-mediated endocytosis, which is limited by the physical size of caveolar invaginations (size cutoff <50 nm) [66,67]. Likewise, targeting receptors associated with clathrin-mediated endocytosis, such as the transferrin receptor, impose an upper size limit for NCs, typically within the range of 80–120 nm [27].

Particle shape can also play a critical role in enhancing vascular endothelial active targeting [46]. Specifically, non-spherical, elongated carriers exhibit more effective binding to the endothelium compared to their spherical counterparts [40,46]. For instance, anti-ICAM1 conjugated nanorods demonstrated up to a threefold increase in binding to static cultured endothelial cells and double the accumulation in the lungs *in vivo* relative to spherical NCs of the same composition [68].

#### Flexibility.

NC flexibility contributes to endothelial targeting through several factors [35,69]. Firstly, flexibility enables larger particles to deform and be internalized *via* endocytic vesicles of small invagination size. For example, flexible PV1 targeted nanogels (150–300 nm) can enter caveolae and accumulate efficiently in the lungs, whereas rigid counterparts of the same size cannot [69,70]. Secondly, flexibility enhances NC endothelial binding efficiency during intense flow [46,71]. Deformable carriers can flatten upon contact with target endothelial surfaces, thereby reducing the drag force exerted by perfusion and high shear stress. By spreading over a larger surface area and engaging more binding ligands, affinity-based NC enhance adhesion strength and decrease the likelihood of shear-induced detachment, improving binding stability under physiological flow conditions [46]. For example, PEG-based hydrogel NCs conjugated to anti-ICAM1 antibodies demonstrated ~50 % higher *in vivo* lung accumulation when formulated as softer particles compared to their rigid counterparts [72]. Similarly, a microfluidic study showed that softer disc NCs exhibited significantly greater binding to adhesive surfaces under the same flow conditions as rigid particles [73].

#### Hemodynamics.

Strong hydrodynamic shear forces can detach NCs bound to the endothelial surface, indicating that endothelial binding is inversely correlated with shear stress [74,75]. In a microfluidic chamber study under varying shear rates, antibody-conjugated NCs exhibited a 50 % reduction in binding to an endothelial monolayer when the shear rate increased from a physiological level (~150 s^−1^) to a high shear condition (~900 s^−1^) [75]. This observation aligns with vascular physiology, as shear stress in capillaries is relatively low (around 1 dyne/cm^2^) compared to larger arteries (up to 100 dyne/cm^2^) [76,77]. The lower shear environments in the micro-vasculature reduce detachment forces acting on targeted carriers, thereby facilitating more stable adhesion and improving the likelihood of successful endothelial targeting [46].

NC geometry can also significantly influence endothelial binding under different shear conditions [35,78,79]. This is demonstrated by *in vitro* flow chamber experiments, where spherical polystyrene particles ranging from 100 nm to 10 μm, coated with anti-*E*-selectin, were assessed for their ability to bind to endothelial surfaces under varying shear forces [80]. At a moderate shear rate (200 s^−1^), specific binding increased with particle size, with larger particles (500 nm to 10 μm) showing greater adhesion [80]. However, at higher shear rates (*e.g*., 1500 s^−1^), the largest particles (5 μm and 10 μm) exhibited reduced binding compared to smaller spheres [80]. This reduction is likely due to the stronger drag forces acting on larger particles at high shear, which can overcome the adhesive interactions and prevent stable attachment to the endothelium.

#### 2.2.1. Active lung targeting and intracellular delivery —Advantages and limitations

Compared with passive delivery, active targeting of DDS achieves much higher cell- and tissue-level selectivity, which can be optimized by fine tuning ligand properties such as density, affinity, and avidity. For example, while increasing ligand density can enhance carrier binding through multivalent interactions, excessively high densities may lead to “ligand overcrowding,” which can impair binding efficiency *via* the disruption of optimal ligand orientation and alignment [81,82]. In mice, deliberately reducing anti-ICAM density on ~150-nm carriers increased selectivity for inflamed lungs compared with high-density coatings that bind to both the healthy and inflamed endothelium [66]. In some cases, high-affinity ligands may hinder tissue penetration by binding too strongly at the vascular surface, preventing deeper access to target tissues [83]. For example, anti-transferrin receptors with high affinity show robust endothelial binding but poor transcytosis [84]. Therefore, empirical optimization of ligand density is often necessary for effective targeted drug delivery [53]. Also, increased ligand density, especially of mAbs conjugated to NC surfaces, can increase immune clearance and activate complement cascades [85].

For intracellular delivery, affinity-based active targeting enables DDS to leverage natural endocytic pathways of endothelial cells, allowing for more precise intracellular trafficking of therapeutic cargo (Fig. 3). For instance, NCs conjugated to ligands associated with caveolae-mediated endocytosis have up to 172-fold enhanced lung targeting compared to untargeted nanocarriers, resulting in highly localized therapeutic effects [86]. Similarly, liposomes targeted to *E*-selectin, which is internalized *via* clathrin-mediated endocytosis, demonstrated efficient endothelial uptake [87,88]. Interestingly, endothelial cells do not usually internalize free antibodies against PECAM1 or ICAM1 [89,90]. However, when these antibodies are presented on multivalent carriers, internalization can occur *via* CAM-mediated endocytosis, which is governed by distinct cellular signaling mechanisms [59,91–93]. This route induces transient cytoskeletal remodeling required for endothelial internalization of ICAM1–targeted NCs and is also sensitive to carrier geometry [59,91,94].

Overall, having a strategically engineered DDS is essential for endothelial targeting. For this reason, fine tuning the physical attributes of NCs allows researchers to enhance the biodistribution, transcytosis efficiency, and targeting of their DDS, making this a critical consideration for successful endothelial targeting.

### 2.3. Targeting the pulmonary endothelium: drug delivery strategies in diseased states

Under pathological conditions, the endothelium becomes severely dysregulated, leading to several molecular and structural changes. Predominantly, certain surface markers are upregulated when the endothelium is inflamed. Notably, ICAM1, vascular cell adhesion molecule-1 (VCAM1), PV1, and *E*-selectin show up to three-fold upregulation on the surface of endothelial cells in inflammatory conditions, helping recruit leukocytes [95–97]. At the same time, expression of immune receptors, such as TLRs and receptors for advanced glycation end products (RAGEs) increase to signal for innate immunity [98]. The selective upregulation of these surface proteins makes them targets for DDS in pathological conditions. And while not significantly upregulated during pathological conditions, there are other surface proteins, including the anticoagulant thrombomodulin (TM), that are expressed in abundance on the surface of ECs of the microvasculites, making them favorable targets as well [99].

**Fig. 4.**
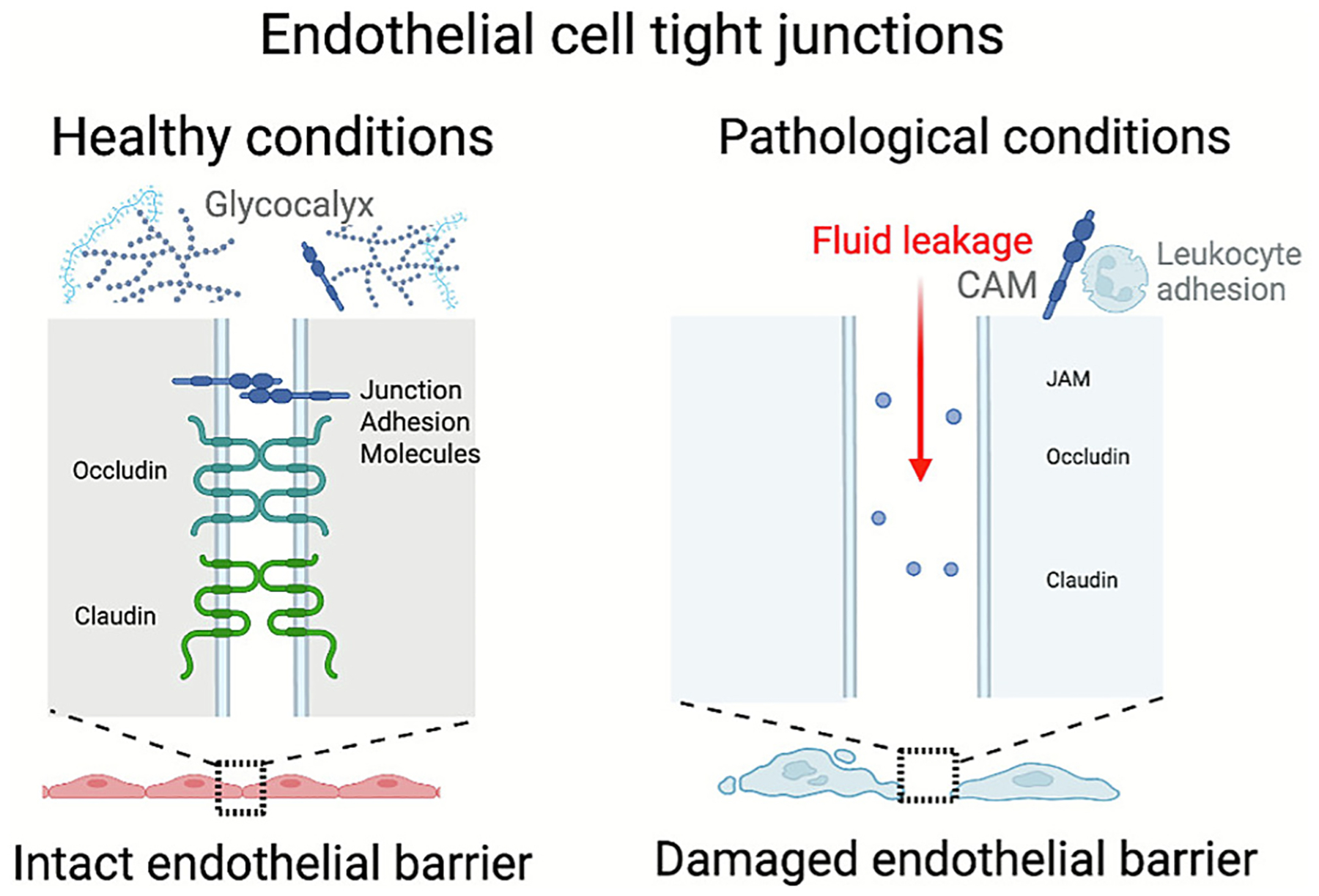
Characteristics of the healthy vs pathological endothelium: Under pathological conditions, the endothelium becomes severely dysregulated, leading to the loss of tight junction integrity and shedding of glycocalyx, making ECs more accessible for DDS. Created in BioRender. Brenner, J. (2025) https://BioRender.com/u6cl422

The degradation of glycocalyx on the surface of endothelial cells also makes proteins more accessible when the endothelium is dysregulated. Glycocalyx is a carbohydrate-rich layer that lines the surface of ECs across the entire vasculature [100]. It consists of a complex network of membrane-bound proteoglycans and glycoproteins which, in totality, form a 1–5 μm negatively charged layer on the surface of the endothelial lumen [101]. Normally, this physical coverage restricts the binding of DDS to adhesion and signaling molecules on the surface of ECs [102]. However, in pathological states, the glycocalyx coat is largely degraded and shed from the vasculature to allow for increased neutrophil adhesion [103]. Inadvertently, this also increases exposure of EC surface proteins for actively targeted DDS (Fig. 4).

Despite an upregulation in the expression of CAMs and other adhesion molecules, the pulmonary endothelium suffers from a loss of tight junction integrity when dysregulated [104]. Inflammation and infections have been shown to induce the degradation of essential tight junction proteins, including claudin-5 and occludin [105]. This makes the lung micro-vascular network susceptible to pathological interstitial and alveolar edema manifested by swelling of the corresponding compartments of the lung tissue [106]. From a drug delivery standpoint, however, the pathologically leaky pulmonary vasculature is more permeable to large biotherapeutic molecules and DDS, not unlike the enhanced permeability and retention (EPR)-like effects typical of abnormal vascular leakiness in solid tumors and other edematous pathologies (Fig. 4) [107].

### 2.4. Pathologies of the lung: acute lung injury, acute respiratory distress syndrome, and pulmonary fibrosis

These defining characteristics of the dysregulated endothelium can be exploited to develop targeted DDS for the treatment of lung pathologies. One of the best-studied conditions for pulmonary drug delivery is Acute Lung Injury (ALI), and its most severe form, Acute Respiratory Distress Syndrome (ARDS). ARDS is a severe, life-threatening condition caused by injury to either pulmonary capillaries or alveoli that prevents sufficient oxygenation and stiffens the lungs [108]. There are numerous risk factors that can trigger the onset of ALI and ARDS, such as pulmonary infection or pneumonia, sepsis, severe trauma, inhalation of toxic fumes, or pancreatitis [109]. Whatever the cause, patients suffering from ALI experience many of the same symptoms, including lung inflammation, endothelial and alveoli apoptosis, edema, hypoxia, accumulation of harmful ROS, and ultimately lung failure [108]. All in all, there are around 70 cases of ARDS per 100,000 people in the US, with an estimated mortality rate of approximately 43 % [109,110]. Despite its severity, there are currently no FDA-approved pharmacological treatments specifically designed for ALI/ARDS [111]. Instead, standard care relies heavily on mechanical ventilation to support oxygenation. However, this may cause ventilator-induced lung injury (VILI), further complicating the disease.

Pulmonary fibrosis (PF) is another chronic lung disease that results in a dysregulated lung endothelium. PF typically originates in the lung interstitium in response to factors such as genetic mutations, bacterial infections, aging, and pollution, which trigger inflammation and fibrosis within the alveolar walls [112]. This directly damages the alveolar-capillary barrier, resulting in vascular remodeling, ECM deposition, and severe damage to endothelial cells and the basal membrane [113]. While PF varies in severity from case to case, once fibrosis begins there is no cure, although there are treatments available that can slow progression and prevent respiratory failure [114].

Given the pathological hallmarks of PF and ALI, particularly endothelial dysregulation and increased expression of adhesion molecules, scientists are engineering DDS targeted to the inflamed pulmonary endothelium, thereby opening new avenues for more precise and effective treatments (Table 1).

#### 2.4.1. Targeting surface proteins on dysregulated ECs: Active Nanocarrier strategies

One notable drug delivery strategy leverages the upregulation of ICAM1 as an accessible target for antibody-functionalized nanoparticles. Liposomes conjugated to anti-ICAM1 antibodies were shown to preferentially accumulate in inflamed pulmonary tissues in a murine model of ALI [115]. Therefore, anti-ICAM1 liposomes are a promising way to enhance localized drug delivery and reduce off-target effects. Expanding this strategy to other NCs, ICAM1-targeted lysozyme-dextran nanogels encapsulating dexamethasone (DXM), a potent anti-inflammatory corticosteroid, significantly reduced lung inflammation and injury in a lipopolysaccharide (LPS)-induced mouse model of ALI [116]. Likewise, DXM-loaded nanostructured lipid carriers functionalized with ICAM1 antibodies have achieved similar therapeutic outcomes [117].

Additionally, several other endothelial ligands have been targeted in ALI-focused DDS strategies. PECAM1-targeted lipid NCs delivering bardoxolone methyl (BM), a triterpenoid with antioxidant and anti-inflammatory properties, was shown to effectively localize to the pulmonary endothelium and mitigate oxidative injury in an *in vivo* ALI model [120]. Beyond lipid NCs, antibody-drug conjugates (ADCs) of anti-PECAM1 antibodies linked to either catalase or superoxide dismutase (SOD) enzymes significantly reduced oxidative stress and improved outcomes in mice [119].

**Fig. 5.**
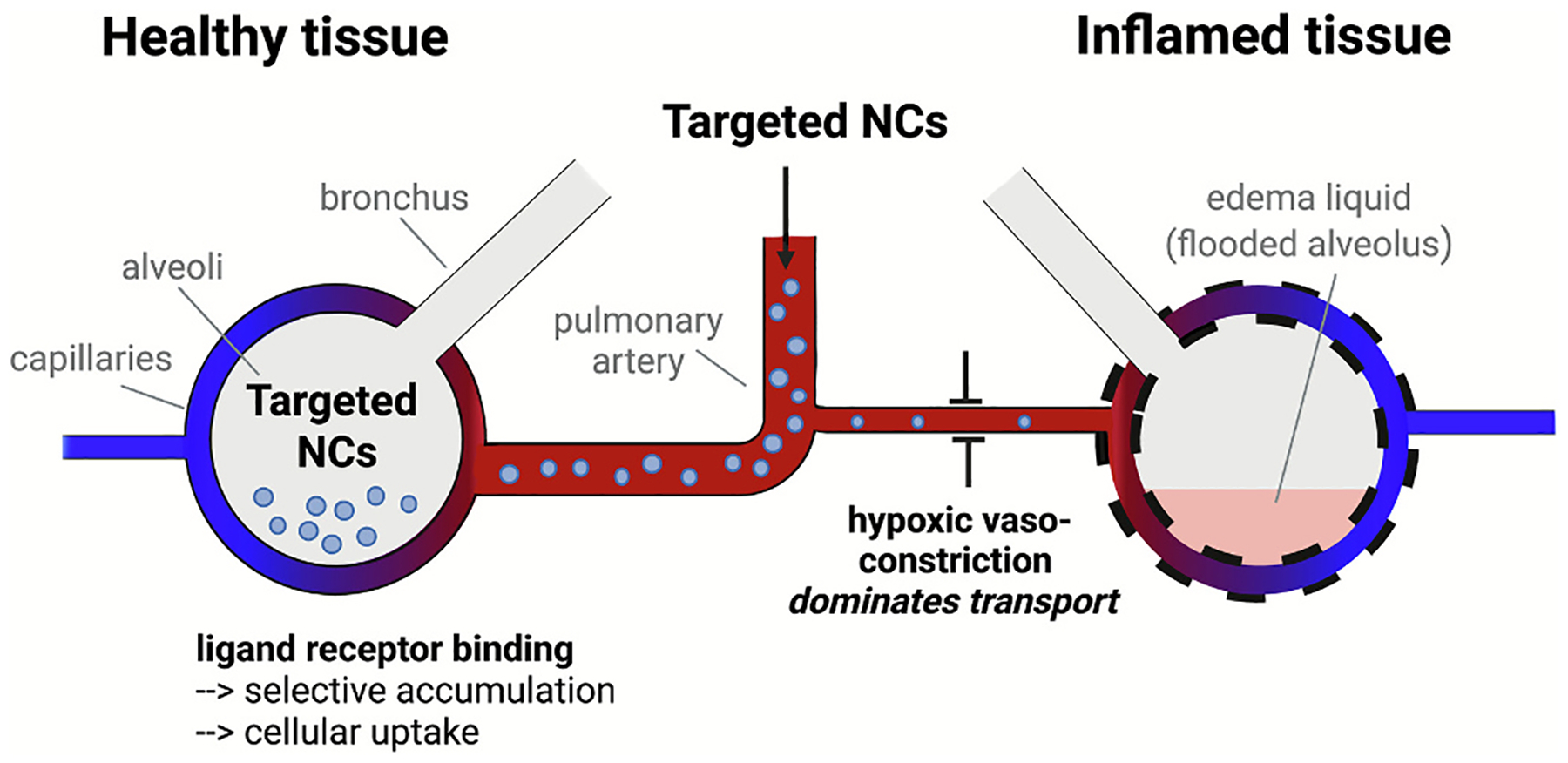
The distribution of targeted DDS, such as anti-PECAM1 conjugated NCs, is determined by relative blood flow to different regions of the lungs. Hypoxic vasoconstriction shunts these targeted NCs away from the dysregulated endothelium in the lung, resulting in preferential accumulation in healthy lung tissue [115]. Created in BioRender. Brenner, J. (2025) https://BioRender.com/u6cl422

Interestingly, a comparison between ICAM1 and PECAM1 targeting reveals that, while PECAM1-targeted antibody-SOD conjugates have superior whole lung uptake, anti-ICAM1 SOD conjugates more effectively mitigate inflammatory signaling in LPS administered mice [64]. This is true for all NCs; PECAM1 targeted liposomes have better whole lung uptake in spatially uniform ARDS mouse models, while ICAM1 targeted liposomes achieve higher local delivery in inflamed tissue. This is because inflamed lungs suffer from hypoxic vasoconstriction, which shunts PECAM1 targeted NCs away from the dysregulated endothelium (Fig. 5). At the same time, there is an increase in ICAM1 surface protein expression in pathological, ARDS-affected regions of the endothelium, promoting localized delivery of ICAM1 targeted NCs [115]. These findings underscore the importance of accounting for spatial heterogeneity in drug delivery and highlight the need to tailor DDS design to effectively target specific regions of pathological endothelium.

Notably, new studies have revealed that some “endothelial targeting” antibodies, including PECAM1 and even more so ICAM1, don’t exclusively deliver their cargo to endothelial cells. Flow cytometry tracking of liposomes conjugated to either anti-ICAM1 or anti-PECAM1 mAbs revealed that uptake in the lungs is roughly equally divided between endothelial cells and marginated neutrophils in the pulmonary vasculature [133]. Marginated neutrophils appear to recognize complement opsonins and the Fc portion of the targeting antibody, subsequently phagocytosing targeted NCs bound to endothelial surfaces before they can be endocytosed [133,134]. This phenomenon helps explain both the increased lung uptake of ICAM1- and PECAM1-targeted DDS and the influence of the targeting moiety on cell-type specificity. For instance, replacing full-length ICAM1 antibodies with Fab fragments lacking the Fc region markedly reduces uptake by marginated neutrophils, making NCs more endothelial-specific [133].

Beyond CAMs, angiotensin-converting enzyme is another widely studied target for endothelial drug delivery. However, in response to inflammation, ACE is shed from ECs, leading to decreased surface expression [135,136]. This impairs the ability of anti-ACE mAbs to target the pulmonary endothelium [137,138]. In a rat model of LPS induced ALI, the delivery of radiolabeled anti-ACE mAbs dropped by 50 % in inflamed *versus* healthy lung tissue. Therefore, AC*E*-targeted DDS are overall less effective in inflammatory conditions. Despite this, ACE-targeted DDS may still have some merit as a prophylactic therapy. As a notable example, pretreating rats with anti-ACE mAbs conjugated to catalase markedly reduced oxidative damage in lung ischemia–reperfusion injured rats compared to controls [124]. However, for therapeutic applications, other targets are likely to be more suitable.

Some other prominent endothelial surface markers that have promise for endothelial targeted DDS are TM, *E*-selectin, and PV1. A variety of TM-targeted DDS, including liposomes containing the cationic polymer N-terminal modified poly(L-lysine) conjugated to anti-TM mAbs, have enhanced delivery to endothelial cells [123]. However, their efficacy in *in vivo* ALI models have yet to be studied. This is because, similar to ACE, TM is shed from the endothelium in pathological conditions, including ALI [118]. For this reason, TM targeted DDS may not be as effective for ALI and other inflammatory pathologies. In contrast, E-selectin is upregulated on the surface of ECs during inflammation, making it a promising target. This is demonstrated by DXM-loaded liposomes conjugated to anti-E-selectin mAb, which alleviate VILI *in vivo* [125]. Similarly, conjugating the anti-fibrotic agent prostaglandin E2 (PGE2) to PV1-targeted antibodies enabled effective endothelial cell delivery and reduced collagen deposition in a PF mouse model [126].

#### 2.4.2. Advanced nanocarrier design: biomimetic and stimuli-responsive approaches to target the dysregulated endothelium

An alternative to directly targeting surface proteins through antibody-NC conjugation is the use of biomimetic membrane-coated drug delivery systems. This strategy aims to mimic the surface properties of immune cells to enhance targeted delivery and avoid clearance, as cloaking nanoparticles in natural cell membranes provides them with the surface antigens and homing capabilities of the parent cell they are trying to emulate [139]. In a notable example, coating poly (lactic-*co*-glycolic acid) nanoparticles loaded with TLR4 siRNA (si-TLR4) in neutrophil membranes was shown to be an effective strategy to deliver RNA therapeutics to inflamed lung tissue, leading to decreased cytokine production [127]. Similarly, macrophage coated mesoporous polydopamine nanoparticles loaded with peimine, an anti-inflammatory steroidal, demonstrated excellent antioxidant activity and significant anti-inflammatory effects *in vivo* [128]. Membrane coating can also be combined with ligand targeting, as DXM-loaded polymeric nanoparticles coated with cell membranes expressing the integrin VLA-4, which binds to VCAM1, enhanced targeting to inflamed lungs and abrogated inflammation in LPS treated mice [121].

Another emerging approach in pulmonary drug delivery is the development of stimuli-responsive NCs. These DDS leverage the pathological microenvironment of the inflamed lung to strategically release their cargo at the affected site. For example, in the majority of ALI cases, neutrophil activation drives the production and release of free radicals and ROS, such as superoxide and hydrogen peroxide [140]. In response, scientists have engineered ROS sensitive DDS. Notably, researchers have designed DXM nanoparticles fabricated with poly(thioketal) bonds which are cleaved by ROS, enabling DXM release at sites of ALI in a murine model [129]. Similarly, antioxidants can be directly conjugated to the surface of nanoparticles. By grafting antioxidant *n*-acetyl cysteine (NAC) on the surface of polymer nanoparticles, they functioned as a scavenger of damaging free radicals, and were shown to be significantly anti-inflammatory both *in vitro* and *in vivo* [130]. ROS-responsive DDS not only enhance therapeutic specificity through innovative design but also minimize off-target effects and systemic toxicity, therefore optimizing the controlled release of therapeutics.

#### 2.4.3. Targeting the dysregulated endothelium through physicochemical design

Other targeting strategies leverage the inherent physicochemical properties of nanoparticles to enable selective endothelial localization without the need for molecular ligands. A prominent example of this strategy formulates permanently charged lipid nanoparticles (LNPs) with a cationic lipid, 2-dioleoyl-3-trimethylammonium-propane (DOTAP) [141]. DOTAP LNPs exhibit strong electrostatic interactions with the negatively charged surfaces of ECs and target the lung endothelium. This mechanism delivers therapeutic cargo, as DOTAP MC3-LNPs loaded with sPD-L1mRNA, an “off switch” for the immune system, significantly attenuated pulmonary inflammatory responses in ARDS mice [131]. Similarly, lipoplexes formulated with the cationic lipid 1,2-di-O-octadecenyl-3-trimethylammonium propane (DOTMA) loaded with short hairpin RNA (shRNA) against the growth factor TGF-β1 significantly decreased fibrosis and collagen levels in an *in vivo* PF mouse model [132]. Physiochemical targeting can also be combined with active ligand targeting strategies, as conjugating anti-VCAM1 antibodies to cationic “SAINT-C18” liposomes effectively delivered anti-inflammatory siRNA to ECs [122].

Physiochemical approaches in DDS have the advantage of simplicity, bypassing the need for complex ligand conjugation steps while still achieving effective drug localization. Unfortunately, there are also significant adverse effects, as DOTAP LNPs have been shown to induce thrombosis in the lungs and exacerbate pre-existing inflammation [142]. Therefore, their safety and efficacy require further characterization before clinical translation relevant to ALI.

#### 2.4.4. Passively targeting the dysregulated endothelium

Beyond the active targeting strategies already mentioned, there are several features of the pathological endothelium that enhance pulmonary DDS specificity. As stated, the size of a nanoparticle influences delivery significantly. For example, peptide-gold nanoparticles that inhibit TLR4 were found to more effectively prevent inflammation, reduce alveolar damage, and increase overall survival at larger diameters (20 nm compared to 13 nm or 5 nm) in an *in vivo* ALI model [143]. Furthermore, the degradation of glycocalyx and loss of tight junction integrity allows greater access to the pathological endothelium. As an example, Guo et al. show that conjugating the corticosteroid prednisolone to a targeting peptide enhanced delivery to inflamed, but not healthy, lung tissue, thereby mitigating off-target side effects [144]. This highlights how the features of the pathological endothelium, notably glycocalyx degradation and junctional leak, make therapeutic drug delivery more effective.

## 3. Additional considerations and challenges for the engineering of EC targeted DDS

Many challenges face researchers designing DDS targeted to the pulmonary endothelium. For one, although endothelial cells share many similar features, significant heterogeneity across ECs exists through different vascular segments. Endothelial cell surfaces of the pulmonary capillaries differ significantly than the arterioles, and these differences are only amplified in states of endothelial dysregulation [145]. This variability impacts the expression of targetable surface markers, making it essential for bioengineers to tailor their DDS to specific, localized endothelial cells of interest.

Likewise, some pathological changes to the pulmonary endothelium are quite different, and in some cases opposite, to those in the systemic vasculature. For example, blood vessels in ischemic extrapulmonary tissue are typically dilated to compensate for hypoxic effect of reduced or ceased perfusion (hypoxic vasodilation) [146]. In contrast, the pulmonary vasculature contracts in sites of compromised ventilation, such as in the areas downstream of obstructed and occluded blood vessels (hypoxic vasoconstriction) [147]. Doing so deviates blood flow from these areas and better ventilated counterparts, thereby helping to maintain oxygenation of blood and alleviating systemic hypoxia. This phenomenon needs to be accounted for in devising DDS intended to deliver therapeutics to the abnormal pulmonary vasculature.

Additionally, the route of DDS administration is central to its therapeutic success. The lung is a unique organ in terms of drug delivery, since agents can be administered through the respiratory pathway directly in the bronchial tree *via* the tracheal and upper ventilatory segments (nose, pharynx, *etc*.) [148]. These intrapulmonary pathways allow direct delivery into lung tissue and, in some cases, beyond the airspace in the organ, providing entry into the blood perfusing the alveolar capillaries [149]. While we have primarily focused on intravenously (IV) delivered DDS in this review, intratracheal, intranasal, and oral routes of administration have also shown promise. For example, Li et al. significantly mitigated remodeling of the pulmonary vasculature and other hallmark symptoms of pulmonary arterial hypertension (PAH), another serious pulmonary pathology, *in vivo via* intranasally delivered R8-conjugated liposomes loaded with circNFXL1 — a circular RNA downregulated in PAH [150,151].

**Fig. 6.**
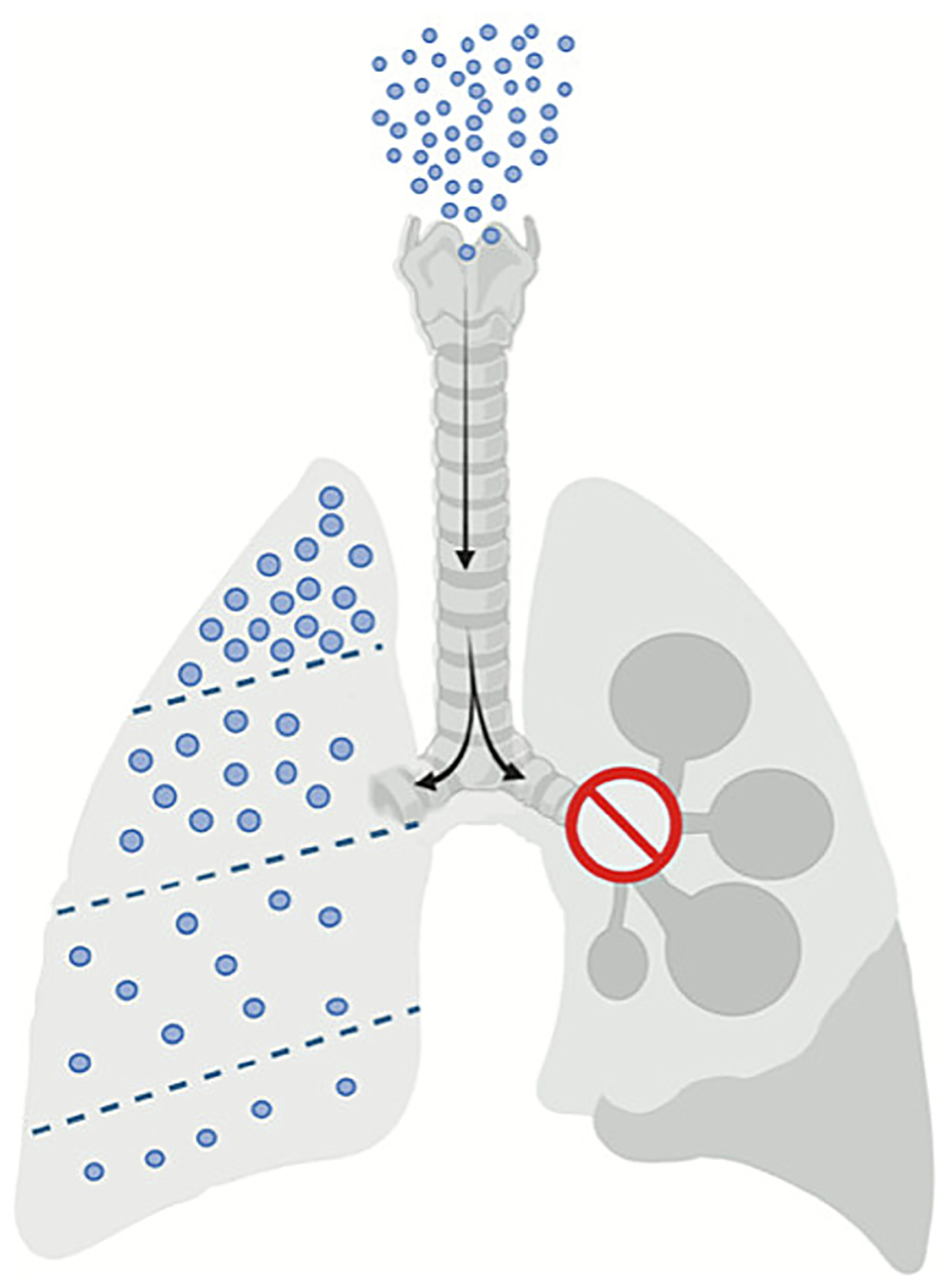
Even under normal circumstances, DDS administered through the airways are heterogeneously delivered across the lungs, as the bifurcations and diameter of bronchi and bronchioles vary in space and time, making the deposition of NCs unpredictable [156]. This is further complicated by pathologies, as abnormalities of the airways will impede nanoparticle delivery, deposition, and distribution across the lobes of the lungs. Created in BioRender. Brenner, J. (2025) https://BioRender.com/u6cl422

However, the capacity of non-IV routes is often suboptimal due to limited or ineffective transport of the DDS in the airways, highly heterogeneous deposition in the tissue, and very poor reach of the alveolar compartments. Intranasal delivery faces anatomical limitations, such as a narrower airway lumen, which is responsible for up to an 85 % drug loss [138]. Intratracheal instillation, commonly used in laboratory settings, enables quantifiable dosing, although it is not physiological, and therefore is limited in its translation to clinical trials. In contrast, aerosol inhalation, used in the clinic, provides more uniform distribution and deeper penetration. However, it is costlier and complicates precise dosing [152]. Moreover, in certain pathological conditions, nanoparticle transport from the airway to the alveoli may be impaired [153]. Notably, in ARDS patients, delivery to alveoli is severely restricted due to edema fluid filling the alveoli and small airways [154,155]. For this reason, in most pathological settings, systemic IV route offers more uniform, effective, fast, safe, and better controlled pulmonary drug delivery (Fig. 6).

Finally, it is essential to highlight safety considerations and possible side effects induced by targeting DDS to the pulmonary endothelium. Several studies have shown that excessive NC uptake by endothelial cells exacerbates EC damage, activates cytokines, and induces inflammation [157,158]. Certain nanoparticles, notably copper and zinc oxide nanoparticles, invoke worse effects. Likewise, lipid nanoparticles damage the endosomal membrane during endosomal escape, which induces LNP associated inflammation (LAI) [159]. As such, it is vital to choose materials that minimize immunogenicity when designing endothelial targeted DDS [142].

## 4. Conclusion

Over the last century, a wealth of research and knowledge has led to huge advancements in endothelial targeted drug delivery. By strategically designing the properties of an NC while exploiting the unique features of the dysregulated endothelium, DDS can be targeted to pathological regions of tissue for efficient therapeutic delivery. Notably, by optimizing the size, shape, rigidity, and ligand characteristics (*e.g*., affinity, density) of an NC, you can design a DDS that is ideal for EC delivery and uptake. Similarly, strategically choosing targeting moieties that are upregulated in inflamed tissue, such as ICAM1, while avoiding those that are shed during pathological conditions (*e.g*., ACE), will ensure that the therapy makes it to affected regions of the pulmonary endothelium.

Despite recent advancements, there are still several challenges and considerations that must be overcome before endothelial targeted drug delivery can become clinically relevant. Notably, nearly all NCs have associated side effects, which only become amplified when they are concentrated in a small region, as is the goal for targeted drug delivery. Therefore, further research needs to be invested into minimizing these adverse reactions. Likewise, targeted DDS are often complex, involving numerous intensive steps to synthesize. While multifaceted DDS are feasible to create in an academic setting, there will be bioengineering challenges when clinically relevant doses of therapies need to be manufactured.

Regardless of these lingering issues, the drug delivery field has seen unprecedented advances over the past couple decades, and will hopefully continue to grow in the years to come as the next generation of scientists tackle these problems.

## Figures and Tables

**Table 1:** Summary of DDS Targeting the Pathological Lung Endothelium.

Drug Delivery System	Targeting Modality	Therapeutic	Disease Model	Efficacy	Ref
Liposome	ICAM1 mAb	N/A	ARDS	Preferentially accumulated in inflamed pulmonary tissue	[115]
Lysozyme Dextran Nanogel		Dexamethasone	Endotoxemia	Suppressed expression of pro-inflammatory CAMs	[116]
Nanostructured Lipid Carrier		Dexamethasone	ALI	Reduced inflammation, attenuated the production of pro-inflammatory cytokines	[117]
Antibody-Enzyme Conjugate		Superoxide Dismutase (SOD)	ALI	More effectively mitigated inflammatory signaling than anti-PECAM-1 conjugated SOD	[118]
Antibody-Enzyme Conjugate	PECAM1 mAb		ALI	Blocked TNF-induced VCAM-1 expression, exhibited superior uptake then anti-ICAM1 conjugated SOD	[119]
Nanostructured Lipid Carrier		Bardoxolone Methyl	ALI	Inhibited the assembly of the NLRP3 inflammasome and pro-caspase-1 complex	[120]
Membrane Coated Polymeric Nanoparticles	VCAM1 mAb	Dexamethasone	ALI	Improved delivery to the inflamed lungs and therapeutic efficacy	[121]
Cationic “SAINT-O-Somes”		NFκB p65 siRNA	ALI	Reduced inflammation	[122]
Cationic Liposomes	TM mAb	N/A	WT Mice	Enhanced delivery to endothelial cells	[123]
Antibody-Enzyme Conjugate	ACE mAb	Catalase	Lung I/R Injury	Prophylactic attenuation of lung injury	[124]
Liposome	*E*-selectin mAb	Dexamethasone	VILI	Reduced symptoms of VILI	[125]
Antibody-Lipid Conjugate	PV1 mAb	Prostaglandin E2	PF	Significantly decreased collagen levels	[126]
Poly (lactic-*co*-glycolicacid) Nanoparticle	Neutrophil Membrane Coating	TLR4 siRNA	ALI	Lowered expression of inflammatory genes	[127]
Mesoporous Polydopamine Nanoparticle	Macrophage Membrane Coating	Peimine	ALI	Inhibited the formation of neutrophil extracellular traps (NETs)	[128]
Polymeric Nanoparticle	Poly(1,4-phenyleneacetonedimethylene thioketal) (PPADT) Cleaved by ROS	Dexamethasone	ALI	Decreased levels of ROS and pro-inflammatory cytokines, and reduced mortality	[129]
Polymer Nanoparticle	N/A	N-Acetyl Cysteine	ALI	Attenuated the number of inflammatory cells, suppressed myeloperoxidase expression, reversed cell apoptosis	[130]
Lipid Nanoparticle	DOTAP	Soluble Programmed Death Ligand-1 mRNA	ARDS	Reduced leukocyte chemotaxis and protein accumulation in the lungs, decreased pulmonary edema, improved survival rate.	[131]
Lipopolyplexes	DOTAM	TGF-β1 shRNA	PF	Significantly decreased hydroxyproline levels and fibrosis	[132]

## Data Availability

No data was used for the research described in the article.
